# Schizandrin Protects Primary Rat Cortical Cell Cultures from Glutamate-Induced Apoptosis by Inhibiting Activation of the MAPK Family and the Mitochondria Dependent Pathway

**DOI:** 10.3390/molecules18010354

**Published:** 2012-12-27

**Authors:** Meng-Shiou Lee, Jung Chao, Jiin-Cherng Yen, Li-Wei Lin, Fan-Shiu Tsai, Ming-Tsuen Hsieh, Wen-Huang Peng, Hao-Yuan Cheng

**Affiliations:** 1 Department of Chinese Pharmaceutical Sciences and Chinese Medicine Resources, College of Pharmacy, China Medical University, Taichung 402, Taiwan; E-Mails: leemengshiou@mail.cmu.edu.tw (M.-S.L.); mthsieh@ mail.cmu.edu.tw (M.-T.H.); 2 Department and Institute of Pharmacology, National Yang-Ming University, Taipei 112, Taiwan; E-Mails: rich720925@yahoo.com.tw (J.C.); jcyen@ym.edu.tw (J.-C.Y.); 3 School of Chinese Medicines for Post-Baccalaureate, I-Shou University, Kaohsiung 840, Taiwan; E-Mails: lwlin@isu.edu.tw (L.-W.L.); asura0734@isu.edu.tw (F.-S.T.); 4 Department of Nursing, Chung Jen College of Nursing, Health Sciences and Management, Chiayi 600, Taiwan

**Keywords:** glutamate, apoptosis, schizandrin, neuroprotection

## Abstract

Glutamate-induced excitotoxicity has been implicated in a variety of neuronal degenerative disorders. In the present study, we investigated the possible neuroprotective effects of schizandrin against apoptosis of primary cultured rat cortical cells induced by glutamate. Glutamate (10 μM) administered for 24 h decreased the expression of Bcl-2 and Bcl-X_L_ protein, whereas increased the expression of Bax, Bak, apoptosis inducing factor (AIF), endonuclease G (Nodo G) and endoplasmic reticulum (ER) stress of caspase-12. Pretreatment with schizandrin (100 μM) before glutamate treatment increased the Bcl-X_L_ and Bcl-2 expression and decreased Bax, Bak, AIF, Nodo G and caspase-12 compared with those only treated with glutamate. Furthermore, glutamate-induced phosphorylation of JNK, p38 and ERK mitogen-activated protein kinases (MAPK), and these effects were attenuated by schizandrin (100 μM) treatment. These results suggest that schizandrin possesses the neuroprotective effects. The molecular mechanisms of schizandrin against glutamate-induced apoptosis may involve the regulation of Bcl-2 family proteins expression, and ER stress through blocking the activation of JNK, ERK and p38 MAPK.

## 1. Introduction

Glutamate (Glu), the principle excitatory amino acid in the CNS, is considered to play an important role in neurotransmission, neuronal development and neurodegeneration, including stroke, ischemia and AD [1]. Glu-induced excitotoxicity, mainly associated with excessive release of Glu and subsequent influx Ca^2+^ via the *N*-methyl-d-aspartate (NMDA)-subtype receptor of Glu, has been implicated in neuronal cell apoptosis [2]. Therefore, searching for the drugs inhibiting apoptosis-induced by Glu is an important step toward the development of effective treatment strategies for neurodegenerative disorder.

There are two main pathways involving apoptotic cell death, one is the interaction of the cell surface receptors, such as Fas and tumor necrosis factor α-1 (TNFα-1) with their ligands, and the second pathway involves the participation of mitochondria [3]. The mitochondrial pathway is regulated by the antiapoptotic and proapoptotic members of Bcl-2 family [4]. The mitochondrial pathway of apoptosis is activated in response to cell death signals such as UV, DNA damage, chemotherapy drugs, ER stress and p53 [5]. Apoptosis is an active process characterized by cell and nuclear shrinkage, chromatin condensation, DNA fragmentation [6], and altered protein expression. The finding that excitotoxic cell death is attenuated by transcriptional and translational inhibitors lends support to an apoptotic mechanism [7]. Indeed, excitatory amino acid-mediated cell death has been shown to regulate the expression of Bcl-2 family proteins in neuronal cultures [8].

Mitochondria play an essential role in apoptosis [9,10]. It is now well established that mitochondria have a critical role in the regulation of apoptosis by acting as reservoirs for a multitude of apoptogenic proteins, such as cytochrome c, Smac/Diablo, Bcl-2 family members, apoptosis inducing factor (AIF), endonuclease G (Nodo G) and caspases [10,11]. The release of these proteins inevitably leads to a wide array of the morphological hallmarks of apoptosis. Bcl-2 family proteins are crucial regulators of apoptosis. On the basis of function and sequence similarity, they can be divided into two groups: the Bcl-2/Bcl-X_L_ subfamily proteins inhibit apoptosis, while the Bax/Bak subfamily promotes cell death [12].

The family of mitogen-activated protein kinases (MAPKs) is important intermediates in signaling pathways that transduce extracellular stimulation into intracellular responses. There are three identified subfamilies of MAPKs: (i) c-Jun *N*-terminal protein kinases (JNK) (ii) extracellular signal regulated protein kinases (ERKs) and (iii) p38 kinases. Recently, certain members of MAPK have been shown to play important roles in neuronal apoptosis in responses to environmental stresses and apoptotic agents [13]. A previous study revealed that abnormal levels of phosphorylated JNK and p38 MAPK in the brain of AD patients is associated with oxidative stress [14]. Other reports have also suggested that ERK and p38 MAPK are involved in Glu-induced cortical cell death and modulate neurodegenerative disease [15,16].

The fruit of *Schisandra chinensis* (Turcz.) Baill. (Schisandrae Fructus) has been used as a kidney and brain tonic in Traditional Chinese Medicine for thousands of years. It possesses many biological properties [17], such as antiamnesic effects [18]. Although the neuroprotective effects of schizandrin have been previously reported [19], little is known about the MAPK activation mechanisms and the role of schizandrin against Glu-induced apoptosis in the mitochondria apoptosis pathway. To further define the molecular mechanism, we used primary culture of rat cortical cells to elucidate the molecular mechanism of schizandrin on the Glu-induced neuronal apoptosis, and the modulations of several cell death proteins were investigated. Dizocilpine, also known as MK801, a well known non-competitive NMDA antagonist [20], has been reported to inhibit Glu-induced neurotoxicity in primary rat cortical cell cultures [21] and was used as a positive control in this study.

## 2. Results and Discussion

### 2.1. Western Blotting Analysis of the Effects of Schizandrin on Glu-Induced Protein Level Changes of Bcl-2 and Bcl-X_L_ in Rat Cortical Cell Cultures

**Figure 1:**
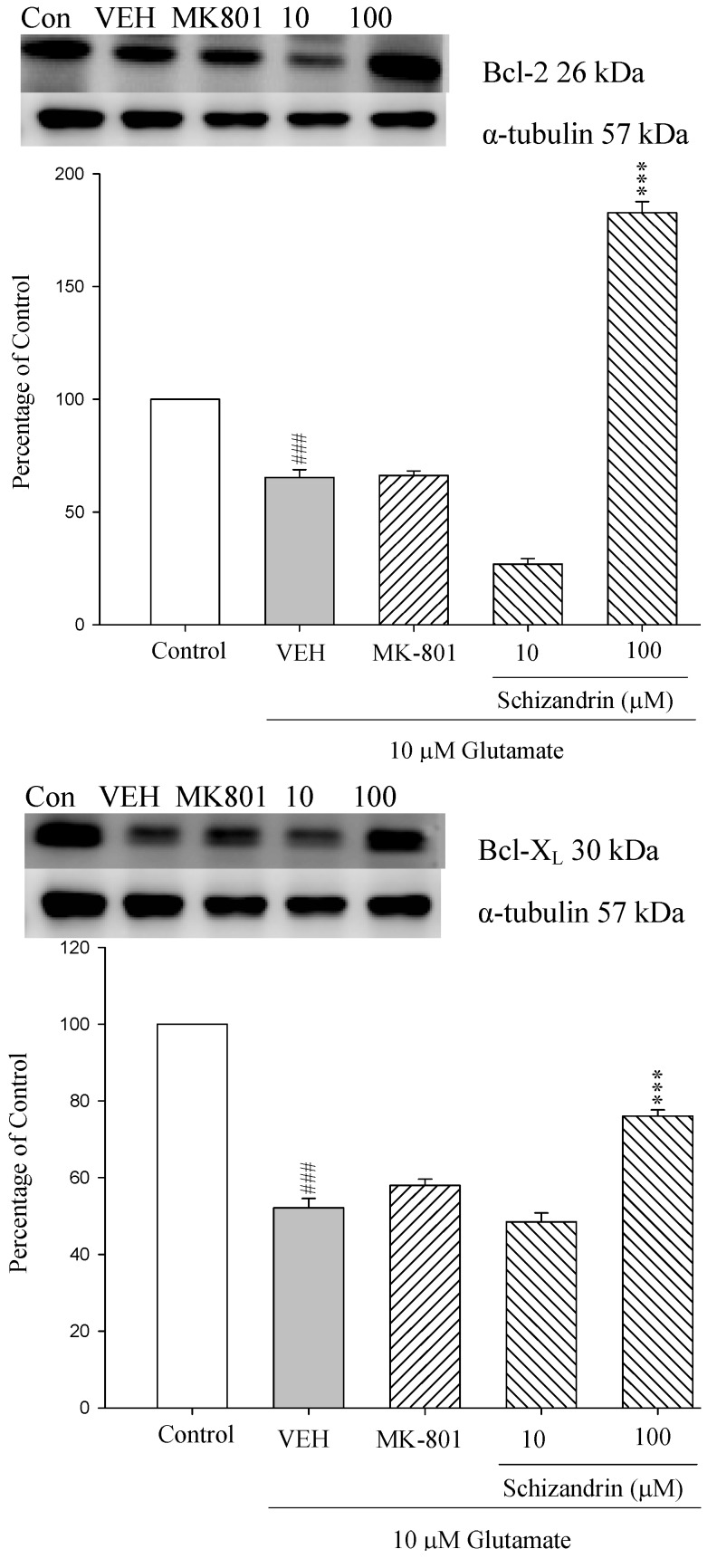
Comparison of western blot analysis of schizandrin on glutamate-induced protein expression level change of Bcl-X_L_, Bcl-2 and α-tubulin in rat cortical cells. Cortical cells were pretreated with schizandrin (10 and 100 μM) or MK-801 (15 μM) 2 h before exposure to 10 μM glutamate and then maintained for 24 h. ^###^ *p* < 0.001 as compared with control group (the protein without glutamate and schizandrin treatment). *** *p* < 0.001 as compared with VEH group (only glutamate treatment). (N = 3). α-Tubulin was used as an internal loading control.

Members of the Bcl-2 protein family act as important regulators and participants in the intrinsic mitochondrial pathway. The release of apoptogenic proteins from the mitochondria is inhibited by anti-apoptotic Bcl-2 family (e.g., Bcl-2 and Bcl-X_L_) [22]. As shown in Figure 1 the protein level of Bcl-2 and Bcl-X_L_ were decreased at 24 h exposure to 10 μM Glu compared with control (*p* < 0.001). Pretreatment of the cell cultures with schizandrin (100 μM) for 2 h significantly increased the protein level changes of Bcl-2 and Bcl-X_L_ compared with those only treated with Glu (*p* < 0.001). These results indicate that schizandrin prevent Glu-induced apoptosis through the protein level of anti-apoptotic Bcl-2 family members in primary cultured rat cortical cells.

### 2.2. Western Blot Analysis of the Effects of Schizandrin on Glu-Induced Protein Level Changes of Bak and Bax in Rat Cortical Cell Cultures

**Figure 2:**
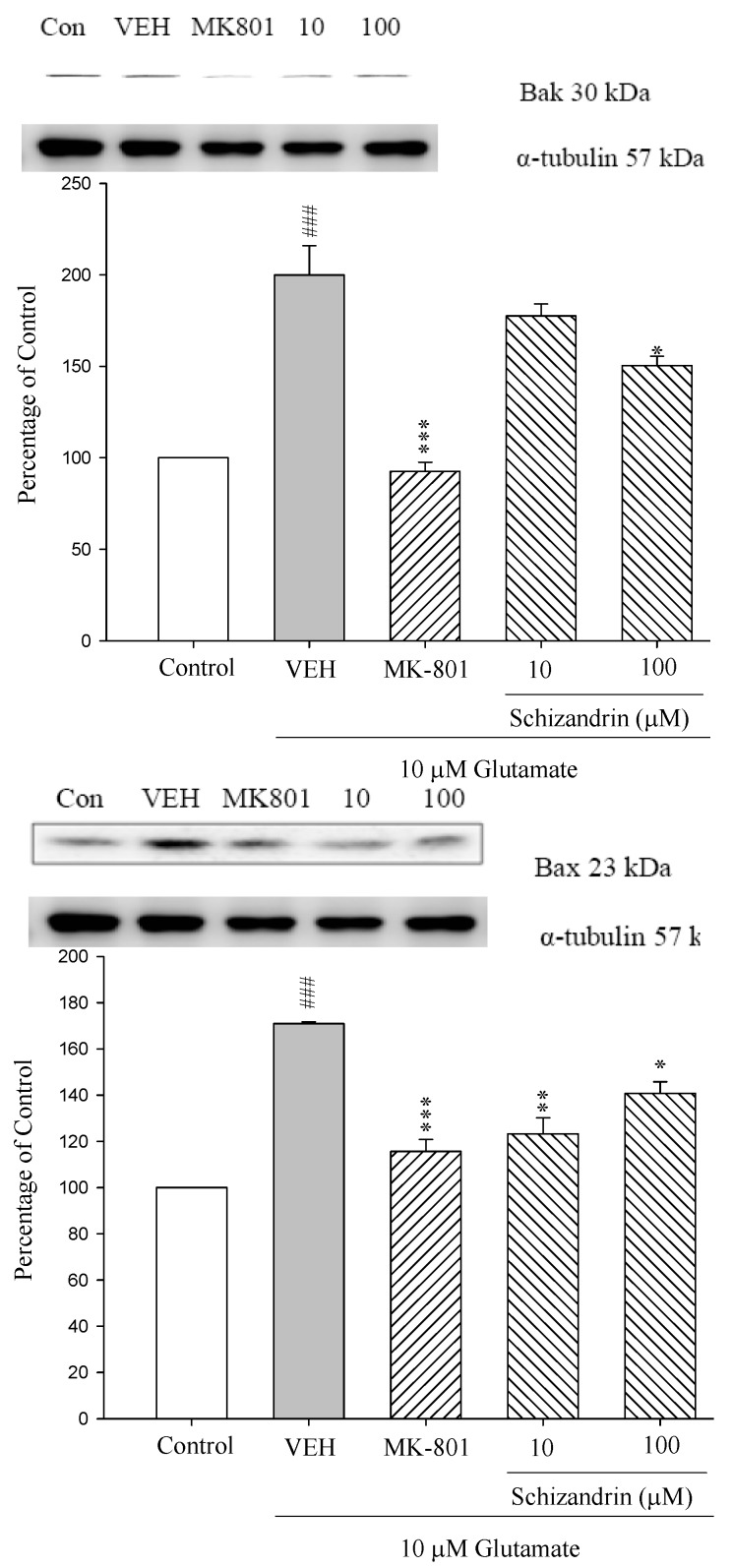
Comparison of western blot analysis of schizandrin on glutamate-induced protein expression level change of Bax, Bak and α-tubulin in rat cortical cells. Cortical cells were pretreated with schizandrin (10 and 100 μM) or MK-801 (15 μM) 2 h before exposure to 10 μM glutamate and then maintained for 24 h. ^###^ *p* < 0.001 as compared with control group. * *p* < 0.05, ** *p* < 0.01, *** *p* < 0.001 as compared with VEH group (N = 3). α-Tubulin was used as an internal loading control.

Pro-apoptotic members of the Bcl-2 family are functionally and structurally divided into two subgroups: multi-domain proteins and ’BH3 domain-only proteins. The most extensively characterized multi-domain pro-apoptotic proteins are Bax and Bak. The activation and oligomerization of Bax and/or Bak leads to disruption of mitochondrial integrity and the release of apoptogenic factors [20]. As shown in Figure 2 the protein levels of Bak and Bax were increased at 24 h exposure to 10 μM Glu (^###^ *p* < 0.001). Pretreatment of the cell cultures with schizandrin (100 μM) and MK-801 (15 μM) for 2 h significantly attenuated the protein level changes of Bak and Bax compared with those only treated with Glu (*p* < 0.05–0.001). Indeed, accumulating evidence suggests that multimers of Bax and Bak form pores that are permeable to cytochrome c [23,24]. This is in agreement with our previous results which indicate schizandrin significantly decreased the cytochrome c release increased by Glu insult [19].

### 2.3. Western Blot Analysis of the Effects of Schizandrin on Glu-Induced Protein Level Changes of Nodo G and AIF in Rat Cortical Cell Cultures

**Figure 3:**
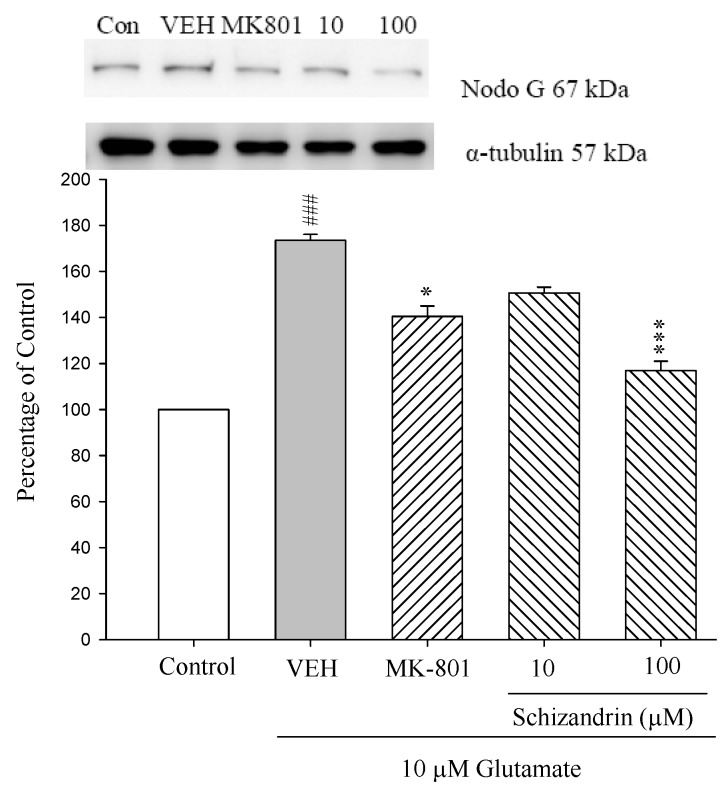

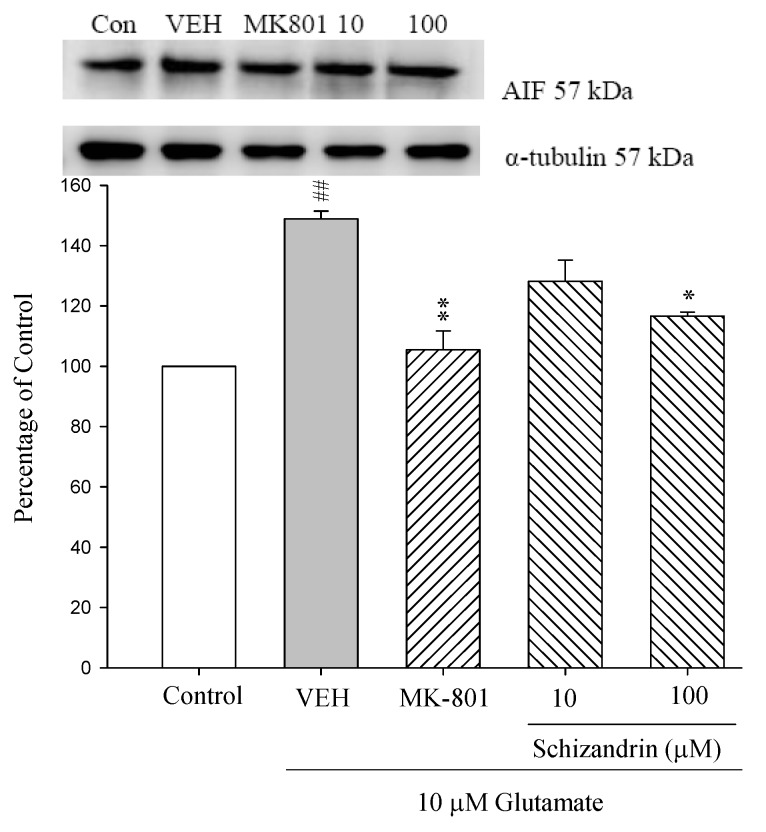
Comparison of western blot analysis of schizandrin on glutamate-induced protein expression level change of AIF, Nod G and α-tubulin in rat cortical cells. Cortical cells were pretreated with schizandrin (10 and 100 μM) or MK-801 (15 μM) 2 h before exposure to 10 μM glutamate and then maintained for 24 h. ^##^ *p* < 0.01 as compared with control group. * *p* < 0.05, ** *p* < 0.01 as compared with VEH group (N = 3). α-Tubulin was used as an internal loading control.

The mitochondrial pathway is initiated by the release of apoptogenic factors such as cytochrome c, apoptosis inducing factor (AIF), Smac/DIABLO, endonuclease G (Nodo G), or caspase-9. The release of cytochrome c into the cytosol triggers caspase-3 activation through formation of the cytochrome c/Apafl/caspase-9-containing apoptosome complex, while AIF and Nodo G promote caspase activation by neutralizing the inhibitory effects to inhibitors of apoptosis proteins (IAPs), have been shown to bind to inhibit caspases [25]. Our previous study indicated that schizandrin attenuated caspase 3 activation by decreasing cytochrome c release. In this study, the protein levels of Nodo G and AIF were increased at 24 h exposure to 10 μM Glu compared with control (*p* < 0.01 and *p* < 0.001). Pretreatment of the cell cultures with schizandrin (100 μM) and MK-801 (15 μM) for 2 h significantly attenuated the protein level changes of Nodo G and AIF compared with those only treated with Glu (*p* < 0.05–0.001) (As shown in Figure 3). In our study, these results indicated that schizandrin and MK-801 prevented Glu-mediated neurotoxicity and this might be partially due to reduced AIF and Nodo G activation and decreased caspase-3 activation.

### 2.4. Western Blot Analysis of the Effects of Schizandrin on Glu-Induced Protein Level Changes of Procaspase 12 and Caspase 12 in Rat Cortical Cell Cultures

**Figure 4:**
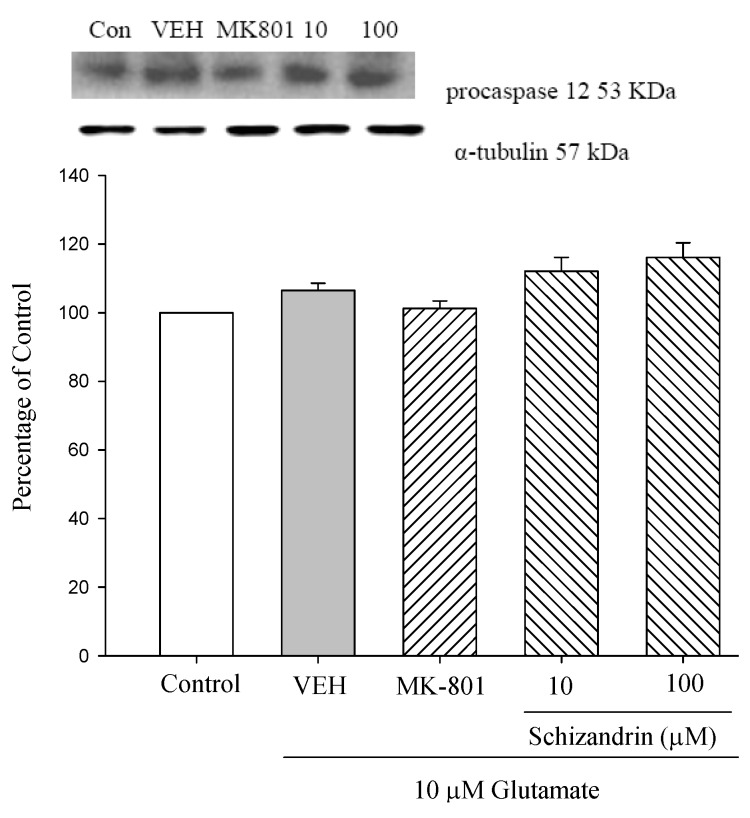

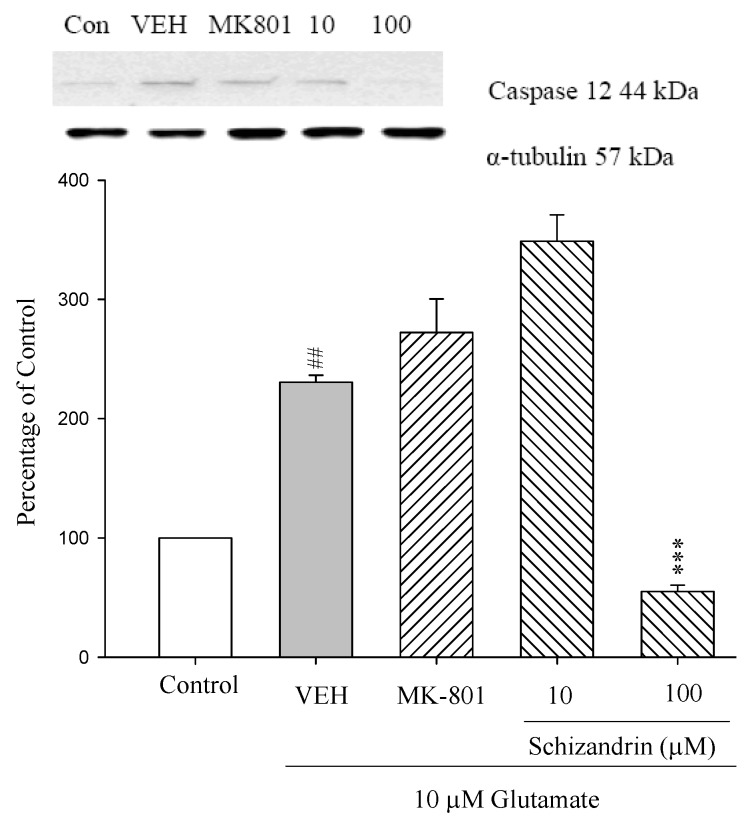
Comparison of western blot analysis of schizandrin on glutamate-induced protein expression level change of procaspase 12, caspase 12 and α-tubulin in rat cortical cells. Cortical cells were pretreated with schizandrin (10 and 100 μM) or MK-801 (15 μM) 2 h before exposure to 10 μM Glu and then maintained for 24 h. ^###^ *p* < 0.001 as compared with control group. *** *p* < 0.001 as compared with VEH group (N = 3). α-Tubulin was used as an internal loading control.

Caspase 12, an endoplasmic reticulum (ER)-specific caspase, participates in apoptosis under ER stress. When ER stress is overwhelming, cells undergo apoptosis, although this was initially reported to be mediated by caspase-12. As shown in Figure 4 both Glu only and schizandrin did not significantly change the level of procaspase 12. Caspase-12 is an ER stress marker and the expression of caspase-12 was significantly increased at 24 h exposure to 10 μM Glu compared with control. Pretreatment of the cell cultures with schizandrin (100 μM) for 2 h significantly attenuated the protein level changes of caspase 12 compared with those only treated with Glu (*p* < 0.001). The data collectively demonstrated that the mitochondrial pathway of apoptosis plays a significant role in ER stress-induced apoptosis.

### 2.5. Western Blot Analysis of the Effects of Schizandrin on Glu-Induced Protein Level Changes of p53 in Rat Cortical Cell Cultures

**Figure 5:**
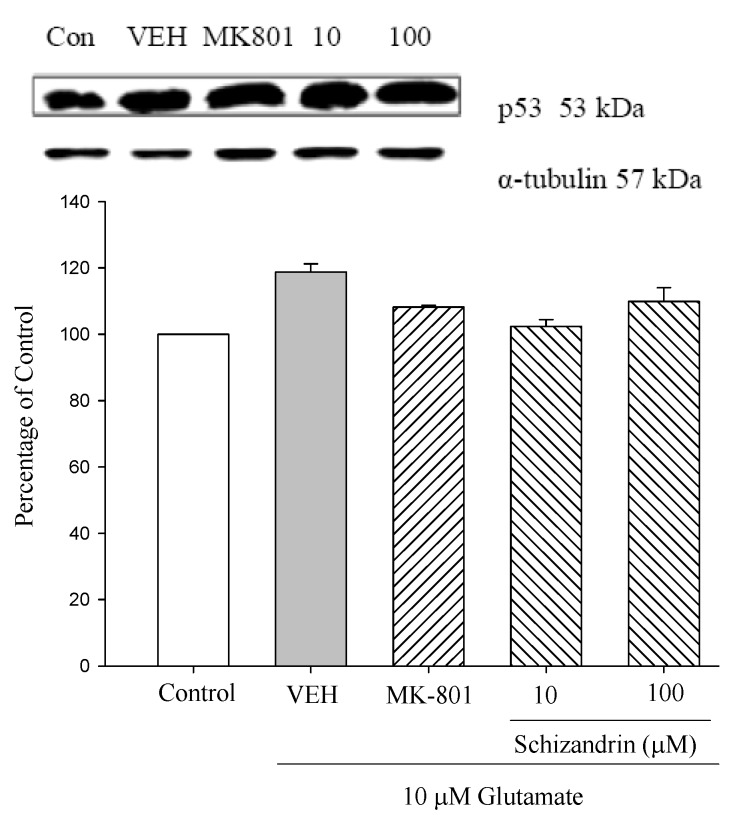
Comparison of western blot analysis of schizandrin on glutamate-induced protein expression level change of p53 and α-tubulin in rat cortical cells. Cortical cells were pretreated with schizandrin (10 and 100 μM) or MK-801 (15 μM) 2 h before exposure to 10 μM glutamate and then maintained for 24 h (N = 3). α-Tubulin was used as an internal loading control.

The tumor suppressor gene p53 has been implicated in the loss of neuronal viability, but the signaling event associated with p53-mediated cell death in cortical neurons is not understood. Previous work has shown that adenovirus mediated delivery of the p53 gene causes cortical neuronal cell death with some features typical of apoptosis. In the present study, we examined the effects of p53 gene on rat primary culture cells. As shown in Figure 5 both Glu only and schizandrin did not significantly change the level of p53. Our study indicate that Glu-induced apoptosis was not dependent on the presence of the p53 gene in primary cultured rat cortical cells.

### 2.6. Western Blot Analysis of the Effects of Schizandrin on Glu-Induced Protein Level Changes of Fas and FasL in Rat Cortical Cell Cultures

**Figure 6:**
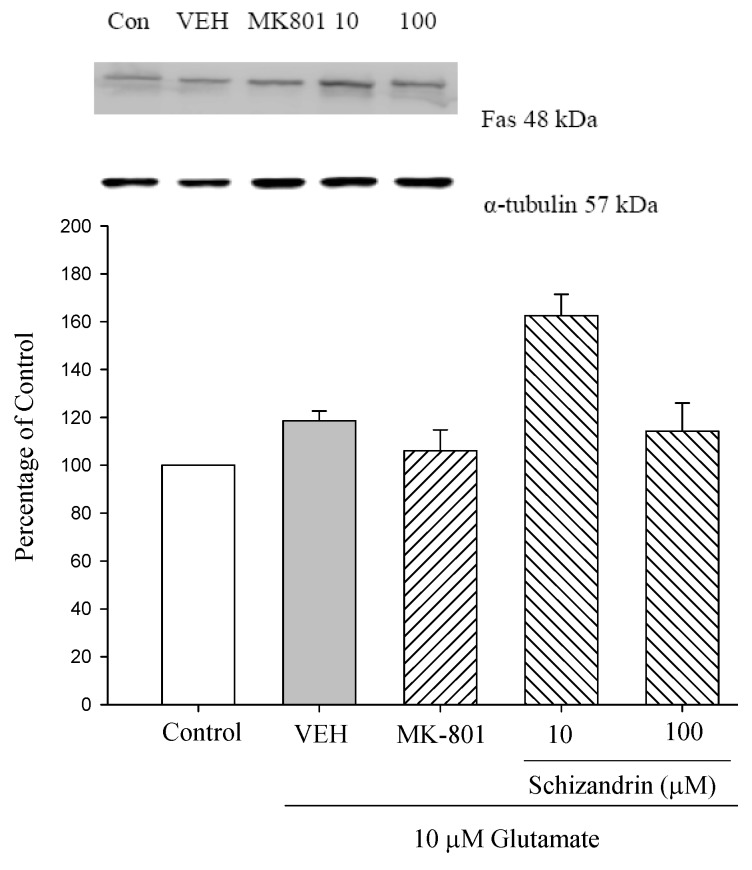

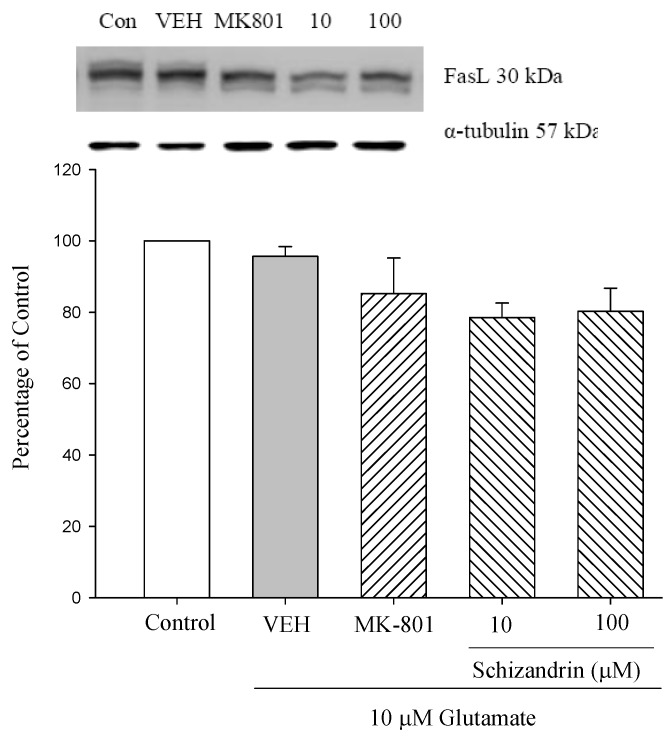
Comparison of western blot analysis of schizandrin on glutamate-induced protein expression level change of Fas and α-tubulin in rat cortical cells. Cortical cells were pretreated with schizandrin (10 and 100 μM) or MK-801 (15 μM) 2 h before exposure to 10 μM glutamate and then maintained for 24 h (N = 3). α-Tubulin was used as an internal loading control.

Apoptosis can be triggered at the cell surface through a receptor-induced signaling pathway (extrinsic pathway), or from within the cell itself via the release of apoptogenic factors, such as cytochrome c from triggered mitochondria (intrinsic pathway) [2]. A typical example of an extrinsic pathway is the activation of a death receptor by the binding of its specific ligand. Some of these death receptors, including TNF-R_l_, Fas and TRAIL-R_l_, contain a preligand binding assembly domain (PLAD) that is located within the first *N*-terminal cysteine-rich domain. Upon binding, death ligands [such as TNF, Fas-ligand (FasL)] reinforce the clustering of their death receptors, which is essential for apoptotic signaling [26]. As shown in Figure 6 neither Glu only or schizandrin produced any significant change to the protein levels of Fas and FasL. This indicates that cell surface receptors, such as Fas and FasL are not involved in this protection mechanism.

### 2.7. Western Blot Analysis of the Effects of Schizandrin on Glu-Induced Protein Level Changes of Phosphor-JNK and α-Tubulin in Rat Cortical Cell Cultures

**Figure 7:**
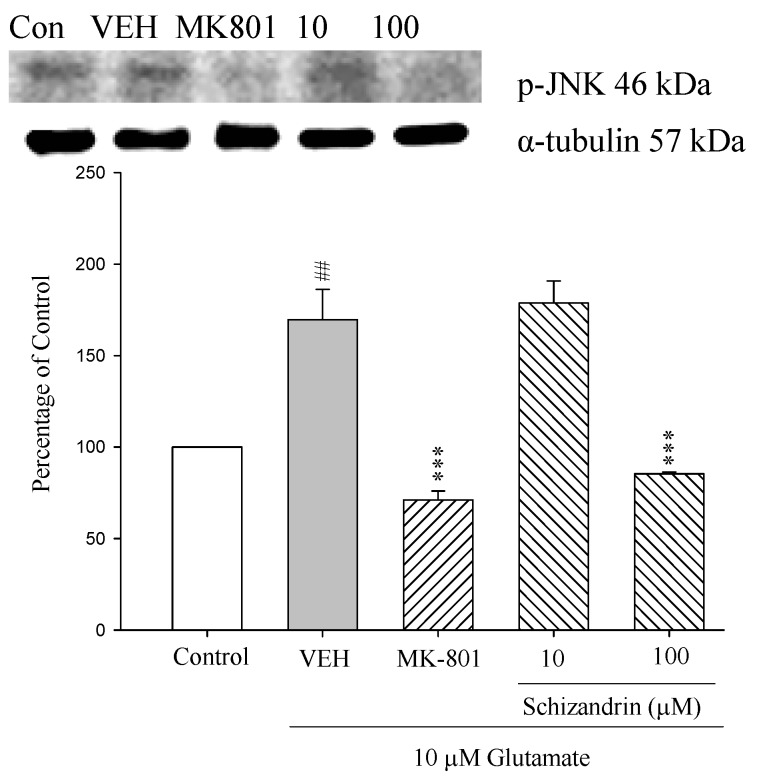
Comparison of western blot analysis of schizandrin on glutamate-induced protein expression level change of phospho-JNK and α-tubulin in rat cortical cells. Cortical cells were pretreated with schizandrin (10 and 100 μM) or MK-801 (15 μM) 2 h before exposure to 10 μM glutamate and then maintained for 24 h (N = 3). ^##^ *p* < 0.01 as compared with control group. *** *p* < 0.001 as compared with VEH group (N = 3). α-Tubulin was used as an internal loading control.

*C*-Jun *N*-terminal protein kinases (JNK, also called stress activated protein kinase), have been shown to play important roles in neuronal apoptosis in response to environmental stresses and apoptotic agents [13]. Previous studies showed that JNK is activated, nuclearly translocated and causally involved in the Glu-induced excitotoxicity [27]. As shown in Figure 7 as indicated by western immunoblot from whole cellular extracts, the activated JNK (p-JNK) was increased at 24 h exposure to 10 μM Glu compared with control (*p* < 0.01). The increase of p-JNK was prevented by schizandrin (100 μM) and MK-801 (15 μM) (*p* < 0.001). These results indicate that schizandrin and MK-801 prevent the Glu-induced apoptosis in rat cortical cells by blocking the phosphorylation of JNK MAPK.

### 2.8. Western Blot Analysis of the Effects of Schizandrin on Glu-Induced Protein Level Changes of Phosphor-ERK in Rat Cortical Cell Cultures

**Figure 8:**
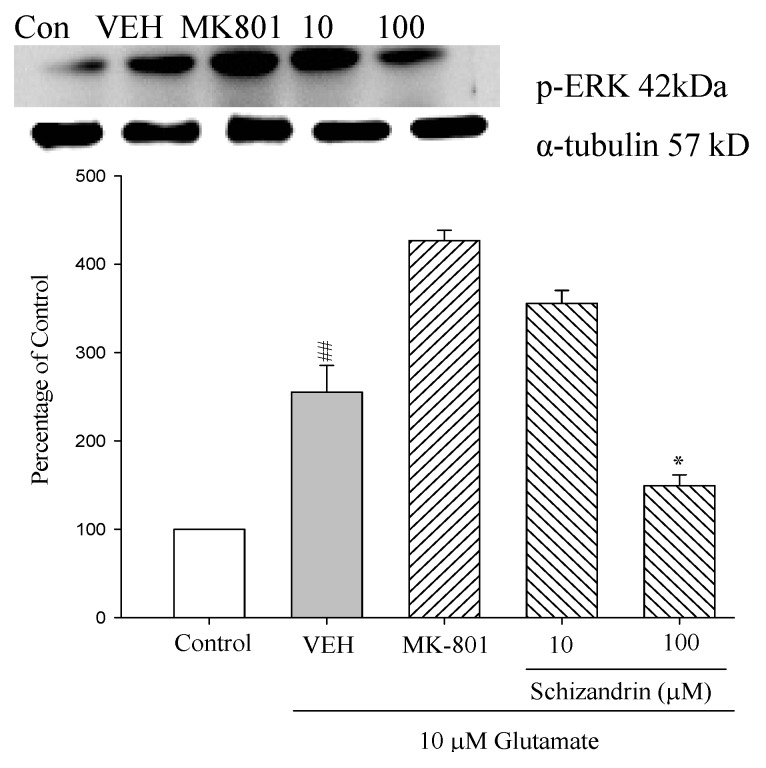
Comparison of western blot analysis of schizandrin on glutamate-induced protein expression level change of phospho-ERK and α-tubulin in rat cortical cells. Cortical cells were pretreated with schizandrin (10 and 100 μM) or MK-801 (15 μM) 2 h before exposure to 10 μM glutamate and then maintained for 24 h. ^##^ *p* < 0.01 as compared with control group. * *p* < 0.05 as compared with VEH group (N = 3). α-Tubulin was used as an internal loading control.

Recent studies revealed that ERK is also activated *in vitro* after relatively mild stimulation of Glu receptors and involved in some physiological events [28]. Furthermore, ERK is found to be activated in some excitotoxicity-associated events such as stroke, seizure and Alzheimer’s disease [29]. In the present study, the protective of schizandrin and the role of ERK in Glu-induced apoptotic-like death in cultured rat cortical neurons were investigated. While the protein level of phosphorylated ERK (*p*-ERK) are remarkably increased after Glu-induction, pretreatment with schizandrin (100 μM) attenuated the p-ERK levels compared with those only treated with Glu (*p* < 0.05) (Figure 8). The results suggest that the activation of ERK is involved in Glu-induced cell death in cultured rat cortical neurons. Our results clearly show that schizandrin attenuated the activation of p-ERK in Glu-treated cortical cells.

### 2.9. Western Blot Analysis of the Effects of Schizandrin on Glu-Induced Protein Level Changes of Phosphorylated-p38 in Rat cOrtical Cell Cultures

**Figure 9:**
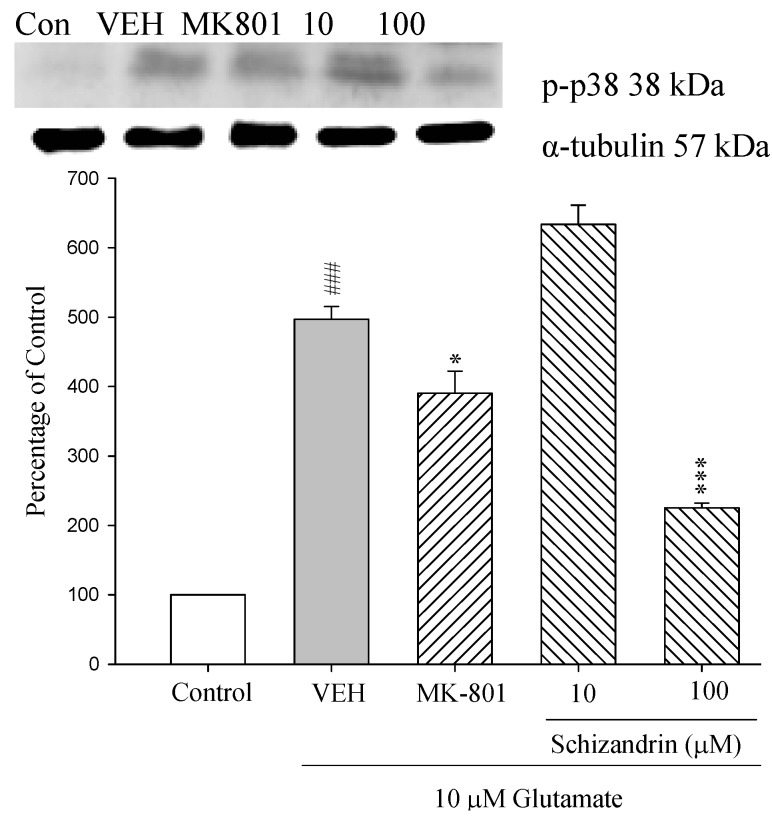
Comparison of western blot analysis of schizandrin on glutamate-induced protein expression level change of phospho-p38 and α-tubulin in rat cortical cells. Cortical cells were pretreated with schizandrin (10 and 100 μM) or MK-801 (15 μM) 2 h before exposure to 10 μM glutamate and then maintained for 24 h. ^###^ *p* < 0.001 as compared with control group. * *p* <0 .05, *** *p* < 0.001 as compared with VEH group (N = 3). α-Tubulin was used as an internal loading control.

The studies indicated that Glu also induces strong activation of p38 and indeed, cell death can be prevented by inhibitors of the p38 pathway [16]. However, little is known about the neuroprotective effect of schizandrin and p38 activation on Glu-induced neuronal death. As shown in Figure 9 the protein level of p-p38 was increased at 24 h exposure to 10 μM Glu compared with control (*p* < 0.001). Pretreatment of the cell cultures with schizandrin (100 μM) and MK-801 (15 μM) for 2 h significantly attenuated the protein level changes of p-p38 compared with those only treated with Glu (* *p* < 0.05 and *p* < 0.001). This study also showed that schizandrin inhibited p-p38 activation to reduce cell death. The protective effects of schizandrin against apoptosis were confirmed by attenuation of p-p38 activation.

### 2.10. Discussion

Apoptosis plays an important role in the development of the nervous system since approximately half of the neurons generated in various regions of the central nervous system (CNS). Glu, the principal excitatory amino acid neurotransmitter in the central nervous system, is also a potent neurotoxin, referred to as excitotoxicity [30]. Our previous study indicated that schizandrin protected primary cultures of rat cortical cells against Glu-induced apoptosis [19]. However, the molecular mechanisms of the action of schizandrin on apoptotic neuronal death induced by Glu have yet to be completely understood.

Apoptosis signaling can be initiated either at the cell surface through a death receptor-induced signaling pathway, or from within the cell itself via the release of proapoptotic factors such as cytochrome c, Bcl-2 family proteins and ER stress from triggered mitochondria. These diverse apoptotic stimuli activate intracellular signals that converge at the level of the mitochondria, resulting in the loss of mitochondrial membrane potential and the release of apoptogenic proteins [31]. There are several types of death receptors in different tissues, but two members of the tumor necrosis factor receptor (TNFR) family and Fas (CD95/Apo-1) were recently demonstrated to be involved in neuronal death [31]. Fas has been extensively studied as a death receptor in lymphocytes. Upon exposure to cell death-triggering stimuli, lymphocytes express at the surface of their membrane the Fas ligand (Fas-L) which binds Fas through an autocrine or paracrine mode. Upon formation of the Fas/Fas-L complex, the Fas-associated death domain (FADD) adaptor protein activates the signaling caspase [32]. As shown in Figure 6 both Glu only and schizandrin did not significantly change the levels of Fas and FasL. It seems also possible that Fas and FasL activation by themselves may not trigger neuronal death by Glu-induced apoptosis.

The Bcl-2 family of proteins has been implicated as a necessary intermediate in the death of a wide variety of cell types [14]. Subcellular localization studies have shown that the anti-apoptotic members of the Bcl-2 family (Bcl-2 and Bcl-X_L_) reside on the mitochondrial outer membrane, while the *pro*-apoptotic family members (Bax, Bad and Bcl-X_S_) may be either cytosolic or present on the cytoplasmic surface of the outer mitochondrial membrane [33]. In the present study, treatment with excitotoxic concentrations of Glu (10 μM) for 24 h resulted in an increase in Bax and Bad protein expressions and a decrease in Bcl-2 and Bcl-X_L_ protein expressions. Pretreatment of the cortical cell cultures with schizandrin (100 μM) for 2 h significantly increased the expressions of Bcl-2 and Bcl-X_L_ protein, whereas it decreased the expressions of Bax and Bak on exposure to 10 μΜ Glu for 24 h. Recent reports have shown that the overexpression of Bcl-2 can protect PC12 cells from oxidative Glu toxicity [34]. Furthermore, a number of reports support Glu receptor-mediated regulation of Bcl-2 and Bax expression in neuronal excitotoxicity [35]. This is in agreement with our previous report that schizandrin (100 μM) and MK-801(15 μM) significantly decreased the cytochrome c release increased by Glu insult [17]. Therefore, these results suggest that schizandrin (100 μM) decreases the cytochrome c release via activating the protein levels of Bcl-2 and Bcl-X_L_ and decreasing the protein levels of Bax and Bak to prevent cytochrome c release.

Another protein that is normally located in the intermembrane space of mitochondria is the apoptosis-inducing factor (AIF). AIF is a flavoprotein which shares homology with the bacterial oxidoreductase and, that, similarly to cytochrome c, is a phylogenetically old, bifunctional protein [36]. Additional DNA degradation occurs due to the release from the mitochondria of AIF and endonuclease G (Nodo G), which constitutes a mitochondria-initiated apoptotic DNA degradation pathway which is conserved between *C*. *elegans* and mammals. Upon death signaling, AIF translocates to the nucleus, binds to DNA and provokes chromatin condensation and large scale DNA fragmentation, apparently in a caspase-independent manner [37]. As shown in Figure 3 and Figure 4, schizandrin (100 μM) and MK-801 (15 μM) for 2 h significantly attenuated the protein level changes of Nodo G and AIF. Therefore, schizandrin and MK-801 may prevent neuronal death by decreasing the level of Nodo G and AIF to avoid the DNA degradation.

P53 (tumor suppressor protein) plays a critical role as a transducer of damage to genomic integrity into growth arrest and/or apoptosis [36]. Several lines of evidence converge to indicate that neurons undergo apoptosis by p53-dependent or -independent pathways, according to the stimulus responsible for stress. Recent reports have shown that NMDA receptor induction upregulates p53 expression [38]. Interestingly, we did not observe a significant increase in p53 expression following exposure to Glu for 24 h. One possible explanation may be that p53 cannot function as a transcriptional activator of Bax or Bad in this model. Taken together with our results, it appears that p53 activation is not required for Glu-induced apoptosis. In addition to the outer mitochondrial membrane (OMM), Bcl-2 and Bcl-X_L_ have also been found to be localized to endoplasmic reticulum (ER) [39]. Since mitochondria are juxtaposed to the ER, in many cases ER stress is communicated to the mitochondria, and ER stress-induced apoptosis is mediated through a dysfunction in the mitochondria. In our study, schizandrin (100 μM) increased the protein level of Bcl-2 and Bcl-X_L_. Similarly, pretreatment of the cell cultures with schizandrin (100 μM) for 2 h significantly attenuated the protein level changes of caspase-12. Taken together with our results findings this clearly supports a role for Bcl-2 family regulation in mediating Glu-induced neurotoxicity.

The major MAPKs have been verified as ERK, JNK, and p38. In addition to apoptosis, other reports have suggested that MAPK pathways may have a protective role in neurons through the inhibition of caspase activation [40]. Previous work in our laboratory suggests that schizandrin may play a protective role in neurodegenerative disease by controlling excitotoxic *pro*-apoptotic pathways involving caspase 3 activation. Moreover, there is also evidence that JNK, ERK and p38 MAPK might mainly act in the nucleus in mediating the Glu-induced excitotoxicity [16,27]. Based on this evidence, we investigated the role of MAPK in schizandrin neuroprotection against Glu-mediated apoptosis. In our study, we showed that Glu caused p-JNK, p-ERK and p-p38 activation in primary rat cortical cells. Thus, our observation that MAPK activation correlated with cytotoxicity and apoptosis is likely to be the more accurate representation of the comparative apoptotic effects of Glu. Furthermore, we also found that pretreatment of neurons with schizandrin-mediated inhibition MAPK activation and neuronal death in response to of Glu toxicity. Thus, our study suggests that schizandrin protects cortical cells from Glu-induced apoptosis by inhibiting activation of p38, ERK and JNK. This neuroprotective effect of schizandrin appears to involve the inhibition of apoptotic damage, possibly through MAPK family-dependent mechanisms.

Schizandrin, a lignan of *Schizandra chinensis*, possesses many biological properties [17], such as antiamnesic effects [18]. In this study we used immunocytochemistry to determine the neuronal marker, MAP-2, and the glial marker, GFAP to assess the viability of the cell culture. Our culture system is neuron-enriched, with 90% of total cultured cells being neurons (data not shown). The present experimental results could express the neuroprotective effect of schizandrin against Glu-induced apoptosis.

It has been reported that Glu and its receptors have long been recognized to play key roles in the pathology of ischemia, leading to the Glu-calcium overload hypothesis [41]. Chiu *et al*. showed that schizandrin B decreases the sensitivity of mitochondria to calcium ion-induced permeability transition (PT) and protects against ischemia-reperfusion injury in rat hearts [42]. Furthermore, schizandrin B pretreatment against CCl_4_ toxicity was paralleled by the decrease in the sensitivity of hepatic mitochondria to Ca^2+^ stimulated PT as well as the attenuations of mitochondrial Ca^2+^ loading. The fruit of *Schizandra chinensis* BAILL, which contains lignans, such as schizandrin, schizandrin B and gomisin A, as main constituents, has been reported to have many biological properties. This is in agreement with our results indicating that the ability for schizandrin to protect primary cultures of rat cortical cells against Glu-induced apoptosis may be related to the resistance of cellular mitochondria to Ca^2+^-stimulated PT. The PT pore was proposed by Haworth and Hunter in 1979 and has since been found to be involved in, among other things, neurodegeneration, a process that results in damage and death of neurons. Induction of the PT pore can lead to mitochondrial swelling and cell death and plays an important role in some types of apoptosis.

## 3. Experimental

### 3.1. Materials and Reagents

Schizandrin was purchased from Wako Pure Chemical Industries, Ltd. (Osaka, Japan). l-Glutamic acid, DNase I, Papain, bovine serum albumin (BSA), and cysteine were purchased from Sigma-Aldrich Chemical Co. (St. Louis, MO, USA). Neurobasal medium, Dulbecco’s modified Eagle’s medium (DMEM), Penicillin, streptomycin and B-27 supplement were purchased from Gibco (Grand Island, NY, USA). Hanks’ balanced salt solution (HBSS) was purchased from Hyclone (Grand Island, NY, USA). MK-801 maleate was purchased from TOCRIS (Ellisville, MO, USA). Antibodies for α-tubulin, Bcl-X_L_, Bcl-2, Bax, Bak, AIF, Nodo G, p53, Fas, Fas ligand (FasL), caspase-12, ERK, JNK, p38, phospho-ERK, phospho-JNK, phospho-p38 and all of the secondary antibodies (anti-rabbit-HRP, anti-mouse-HRP, and anti-goat-HRP) were purchased from Santa Cruz Biotechnology (Santa Cruz, CA, USA). The chemiluminescent reagents were purchased from Billerica immobilon western (Billerica, MA, USA).

### 3.2. Primary Cultures of Rat Cortical Cell

Primary neuronal cultures of cerebral cortex were obtained from rat embryos (E16-18) according to Nishikawa with modifications as described before [26,27]. The cerebral cortex of rat embryos were dissected and placed in cold Hanks’ balanced salt solution (HBSS). After removal of meninges, the tissues were minced and incubated at 37 °C for 15 min in Ca^2+^/Mg^2+^-free HBSS contained 0.25% trypsin and 0.2 mg/mL DNase I, and the cell suspensions were centrifuged (300 g for 10 min). The resulting pellets were resuspended in the 1:1 mixture of DMEM and F12 supplemented with 20% heat-inactivated FBS, 100 U/mL penicillin, and 100 μg/mL streptomycin. Cells were plated into poly-d-lysine coated dishes at a density of 1.5 × 10^5^/mL onto 96 well. Cytosine arabinoside (10 μM) was added to culture 24 h after plating to prevent nonneuronal cell proliferation. Cortical cell cultures were incubated at 37 °C in 5% CO_2_. After 48 h, the medium was replaced with neurobasal medium supplemented with B-27 and penicillin (without l-Glu, Gibco). Only mature cultures (12–14 days *in vitro*) were used for experiments. Immunochemical staining with anti-microtubule associated protein-2 (MAP-2) antibody and anti-glial fibrillary acidic protein (GFAP) antibody revealed that the culture method used in this study provided cell cultures containing about 90% neurons as described previously [27].

### 3.3. Excitotoxicity Induction and Drug Treatment

l-Glutamic acid was dissolved in distilled water at a concentration of 100 μM (final 10 μM). Schizandrin and MK-801 were dissolved in methanol and distilled water and diluted with medium. On the day of the experiment, schizandrin and MK-801 were added to culture medium for 2 h, and then the cells were exposed to 10 μM Glu and maintained for 24 h.

### 3.4. Protein Extraction and Western Blot Analysis

Cortical cells were scraped and washed once with PBS. The cell suspension was then spun down, and the cell pellets were lysed for 30 min in lysis buffer (50 mM Tris (pH 7.5), 0.5 M NaCl, 1.0 mM EDTA (pH 7.5), 10% glycerol, 1 mM basal medium Eagle, 1% Igepal-630, and proteinase inhibitor cocktail tablet) and spun down at 12,000 *g* for 10 min. Then, the supernatants were removed and placed into new Eppendorf tubes. The supernatant was collected and stored at −80 °C for further western experiments. Proteins from the cortical cells were separated on 12% gradient SDS-PAGE and transferred to nitrocellulose membranes. Nonspecific protein binding was blocked in blocking buffer at RT for 1 h (5% milk, 20 mM Tris HCl, pH 7.6, 150 mM NaCl, and 0.1% Tween 20). The nitrocellulose was then incubated with specific antibodies of Bcl-X_L_, Bcl-2, Bax, Bak, AIF, Nodo G, p53, Fas, FasL, caspase-12, JNK, ERK, p38, phospho-JNK, phospho-ERK, phospho-p38 and α-tubulin (Santa Cruz Biotechnology) as indicated for each experiment in the blocking buffer at 4 °C overnight. On day 2, filters were rinsed three times in PBST buffer (PBS+0.1% Tween 20), and then incubated with the corresponding peroxidase-conjugated secondary antibodies (diluted 1:5000) for 1 h at temperature. Blots were developed using the enhanced chemiluminescence (ECL) (LAS-3000mini; Fujifilm, Kanagawa, Japan) detection method by immersing them for 5 min in a mixture of ECL reagents A and B at the ratio 1:1 and exposing them to photographic film for a few minutes. For quantification, the signal intensity on western blots was evaluated with image Gauge version 4.1 (Fuji Film) using the LAS-3000 system.

### 3.5. Statistical Analysis

Data are expressed as mean ± SEM. Statistical evaluation was carried out by One-way analysis of variance (One way ANOVA) followed by Scheffe’s multiple range test. The *P* value less than 0.05 were considered significantly.

## 4. Conclusions

In conclusion, our study demonstrated that the inhibition of mitochondrial translocation of Bcl-2 family and the activation of MAPKs were critical events in the schizandrin protection against apoptosis from Glu-insult of cortical neuron cells. We suggest that schizandrin possesses neuroprotective potential. On the basis of the present study, the anti-apoptotic effect of schizandrin may provide a potential therapeutic approach for preventing and/or treating neurodegenerative diseases. However, is there a yet unidentified mitochondrial receptor for these proteins? Further in-depth studies would be necessary to interpret the mechanisms.
